# Multi-source heterogeneous data fusion and intelligent prediction modeling for chemical engineering construction projects based on improved transformer architecture

**DOI:** 10.1038/s41598-025-22752-2

**Published:** 2025-11-05

**Authors:** Dongfeng Xin

**Affiliations:** Huajin Aramco Petrochemical Company Limited, Panjin, 124000 Liaoning China

**Keywords:** Transformer architecture, Heterogeneous data fusion, Chemical engineering construction, Multi-scale attention mechanism, Intelligent prediction modeling, Multi-task learning, Engineering, Mathematics and computing

## Abstract

This paper presents a comprehensive framework for multi-source heterogeneous data fusion and intelligent prediction modeling in chemical engineering construction projects using improved Transformer architecture with enhanced attention mechanisms. The proposed methodology addresses critical challenges in integrating diverse data modalities including structured numerical measurements, semi-structured operational logs, and unstructured textual documentation through innovative multi-scale attention mechanisms and cross-modal feature alignment modules. Key technical contributions include an adaptive weight allocation algorithm for dynamic data source management and a multi-task learning framework enabling simultaneous progress estimation, quality assessment, and risk evaluation. Comprehensive experimental validation demonstrates prediction accuracies exceeding 91% across multiple tasks, representing improvements of up to 19.4% over conventional machine learning techniques and 6.1% over standard Transformer architectures. Real-world deployment in three major chemical engineering construction projects confirms practical viability with robust anomaly detection capabilities achieving 92% + detection rates and real-time processing performance under 200 ms. The integration of interpretability mechanisms through attention visualization and SHAP analysis provides transparent decision-making processes aligned with engineering domain expertise requirements.

## Introduction

### Background and significance

Chemical engineering construction projects represent complex industrial undertakings that generate vast quantities of multi-source heterogeneous data throughout their lifecycle, encompassing design specifications, material properties, environmental monitoring data, equipment sensor readings, and construction progress records^1^. The integration and intelligent analysis of these diverse data streams have become increasingly critical for enhancing project management efficiency, reducing construction risks, and optimizing resource allocation in modern chemical engineering facilities^2^. However, the inherent complexity of chemical engineering construction environments, characterized by multi-disciplinary collaboration, dynamic project conditions, and stringent safety requirements, presents significant challenges for effective data fusion and predictive modeling.

The growing scale and complexity of chemical engineering construction projects have amplified the importance of intelligent data processing capabilities^3^. Traditional approaches to data management in chemical engineering construction typically rely on isolated systems and manual integration processes, resulting in information silos, delayed decision-making, and suboptimal resource utilization. Furthermore, the heterogeneous nature of data sources, ranging from structured databases to unstructured sensor streams and documentation, compounds the difficulty of achieving comprehensive data integration and analysis.

### Challenges and limitations of traditional methods

Conventional data processing methodologies in chemical engineering construction face several critical limitations that hinder their effectiveness in modern project environments^4^. Traditional statistical methods and rule-based systems often struggle to capture the complex, nonlinear relationships inherent in multi-source heterogeneous data, particularly when dealing with high-dimensional datasets and temporal dependencies. These approaches typically require extensive domain expertise for feature engineering and rule definition, limiting their adaptability to diverse project contexts and evolving construction scenarios.

Moreover, classical machine learning techniques frequently encounter difficulties when processing data with varying formats, sampling rates, and quality characteristics^5^. The lack of unified representation frameworks for heterogeneous data sources results in information loss during integration processes and reduces the accuracy of predictive models. Additionally, traditional methods often exhibit poor scalability and computational efficiency when applied to large-scale chemical engineering construction datasets, constraining their practical applicability in real-world industrial settings.

### Advantages of transformer architecture and attention mechanisms

The emergence of Transformer architecture and attention mechanisms has revolutionized the field of data processing and offers promising solutions for addressing the challenges associated with multi-source heterogeneous data fusion^6^. The self-attention mechanism inherent in Transformer models enables the capture of long-range dependencies and complex interactions between different data modalities without the constraints of sequential processing requirements. This capability is particularly valuable for chemical engineering construction applications, where temporal and spatial relationships between various data sources significantly influence project outcomes.

Transformer-based approaches demonstrate superior performance in handling variable-length sequences and can effectively process heterogeneous data types through unified embedding representations^7^. The parallel processing capabilities of Transformer architectures provide computational advantages for large-scale data fusion tasks, while their attention mechanisms offer interpretability benefits that are crucial for engineering decision-making processes. Furthermore, the scalability and transfer learning capabilities of Transformer models enable adaptation to diverse chemical engineering construction contexts with minimal fine-tuning requirements.

## Research objectives and innovations

This study aims to develop a comprehensive framework for multi-source heterogeneous data fusion and intelligent predictive modeling in chemical engineering construction projects, leveraging the advanced capabilities of Transformer architecture and attention mechanisms^8^. The primary research objectives include establishing unified data representation methods for heterogeneous sources, designing adaptive attention mechanisms for cross-modal information integration, and developing intelligent predictive models for construction progress, resource requirements, and risk assessment.

The key technical innovations of this research represent fundamental advances beyond existing Transformer-based fusion approaches: (1) Domain-specific multi-scale attention mechanism that explicitly models the temporal hierarchies inherent in chemical engineering construction processes, addressing the challenge of processing data streams with vastly different sampling frequencies (millisecond sensor readings to monthly progress reports); (2) Contrastive cross-modal alignment framework that learns semantic correspondences between heterogeneous modalities without requiring manually crafted feature mappings, enabling automatic discovery of relationships between numerical sensor data, textual documentation, and categorical project states; (3) Adaptive weight allocation algorithm that dynamically adjusts data source contributions based on real-time quality assessment and task-specific relevance, addressing the practical challenge of varying data reliability in industrial environments; (4) Multi-task learning architecture specifically designed for simultaneous prediction of interdependent engineering objectives (progress, quality, risk) while maintaining task-specific adaptation capabilities.

These innovations address critical limitations of existing approaches that typically require extensive manual feature engineering, assume static data quality, and process modalities independently without considering domain-specific temporal structures common in chemical engineering construction projects.

### Paper organization

The remainder of this paper is organized as follows: Section II presents a comprehensive literature review of data fusion techniques and Transformer applications in engineering domains. Section III details the proposed methodology, including the Transformer-based data fusion framework and attention mechanism design. Section IV describes the experimental setup and dataset characteristics. Section V presents the results and performance analysis. Section VI discusses the implications and practical applications of the findings. Finally, Section VII concludes the paper and outlines future research directions.

## Related technical and theoretical foundations

### Transformer architecture principles and development

The Transformer architecture, introduced as a revolutionary approach to sequence modeling, fundamentally transformed the landscape of deep learning by replacing traditional recurrent and convolutional structures with a pure attention-based mechanism^9^. The core innovation of the Transformer lies in its self-attention mechanism, which enables the model to capture long-range dependencies and parallel processing capabilities that overcome the sequential limitations inherent in recurrent neural networks.

Recent advances in Transformer-based architectures for time series and multimodal data processing have established strong foundations for industrial applications, yet significant gaps remain in handling the specific challenges of chemical engineering construction environments. Table 1 presents a systematic comparison of existing approaches and highlights the positioning of this research within the current landscape.

**Table 1:** Comparison of Transformer-based Fusion Approaches.

Approach	Data Types	Attention Mechanism	Domain Adaptation	Industrial Validation
Standard Transformer^9^	Homogeneous sequences	Single-scale self-attention	General purpose	Limited
Vision Transformer^10^	Image + text	Patch-based attention	Computer vision	None
TimesFormer^11^	Video sequences	Spatial–temporal attention	Video analysis	None
Industrial BERT^12^	Text + numerical	Pre-trained embeddings	Manufacturing	Simulated data
Proposed Method	Multi-modal heterogeneous	Multi-scale adaptive	Chemical engineering	Real-world deployment

The evolution of attention mechanisms from basic self-attention to specialized variants demonstrates increasing sophistication in handling complex data relationships, yet most existing approaches lack the domain-specific design considerations necessary for industrial deployment in chemical engineering contexts.

#### Encoder-decoder structure and self-attention mechanism

The Transformer architecture adopts an encoder-decoder framework where both components utilize identical structural units composed of multi-head attention layers and position-wise feed-forward networks^13^. The fundamental building block of this architecture is the scaled dot-product attention mechanism, mathematically expressed as:

$${\text{Attention}}\left( {Q,K,V} \right) = {\text{softmax}}\left( {\frac{{QK^{T} }}{{\sqrt {d_{k} } }}} \right)V$$

where $Q$, $K$, and $V$ represent the query, key, and value matrices respectively, and ${d}_{k}$ denotes the dimension of the key vectors. The multi-head attention mechanism extends this concept by computing attention in parallel across multiple representation subspaces:

$${\text{MultiHead}}\left( {Q,K,V} \right) = {\text{Concat}}\left( {{\text{head}}_{1} , \ldots ,{\text{head}}_{h} } \right)W^{O}$$

where each attention head is defined as:

$${\text{head}}_{i} = {\text{Attention}}\left( {QW_{i}^{Q} ,KW_{i}^{K} ,VW_{i}^{V} } \right)$$

#### Positional encoding and structural components

Since the Transformer architecture lacks inherent sequential processing capabilities, positional encoding becomes essential for incorporating sequential information into the model^14^. The sinusoidal positional encoding scheme provides deterministic position representations using trigonometric functions:

$$PE_{{\left( {pos,2i} \right)}} = {\text{sin}}\left( {\frac{pos}{{10000^{{2i/d_{model} }} }}} \right)$$

$$PE_{{\left( {pos,2i + 1} \right)}} = {\text{cos}}\left( {\frac{pos}{{10000^{{2i/d_{model} }} }}} \right)$$

where $pos$ represents the position index, $i$ denotes the dimension index, and ${d}_{model}$ is the model dimension.

The architecture incorporates residual connections and layer normalization to facilitate training stability and gradient flow^15^. Layer normalization is applied according to:

$${\text{LayerNorm}}\left( x \right) = \gamma \frac{x - \mu }{\sigma } + \beta$$

where $\mu$ and $\sigma$ represent the mean and standard deviation computed across the feature dimension, while $\gamma$ and $\beta$ are learnable parameters. The residual connection combines the input and output of each sub-layer:

$${\text{Output}} = {\text{LayerNorm}}\left( {x + {\text{Sublayer}}\left( x \right)} \right)$$

#### Feed-forward networks and computational advantages

Each encoder and decoder layer contains a position-wise feed-forward network that processes each position independently through two linear transformations with ReLU activation:

$${\text{FFN}}\left( x \right) = {\text{max}}\left( {0,xW_{1} + b_{1} } \right)W_{2} + b_{2}$$

This design choice enables efficient parallel computation and maintains the model’s ability to capture complex nonlinear relationships within the sequential data.

#### Applications in time series and multimodal data processing

The Transformer architecture has demonstrated remarkable versatility in time series forecasting applications, where its attention mechanisms effectively capture temporal dependencies without the computational constraints of recurrent processing^16^. The model’s ability to attend to relevant historical information across arbitrary time spans makes it particularly suitable for long-term prediction tasks in engineering applications.

In multimodal data processing contexts, Transformers excel at learning cross-modal representations and capturing interactions between heterogeneous data sources^17^. The attention mechanism naturally accommodates varying input modalities through learned embeddings, enabling unified processing frameworks for diverse data types commonly encountered in chemical engineering construction projects. The scalability and interpretability advantages of Transformer-based approaches have established them as preferred architectures for complex industrial data fusion and predictive modeling applications.

### Theoretical foundations of attention mechanisms

#### Mathematical principles of self-attention mechanism

The self-attention mechanism constitutes the fundamental computational unit that enables Transformer architectures to capture complex dependencies within sequential data through learnable similarity measures^18^. The core mathematical formulation begins with the transformation of input representations into three distinct vector spaces, representing queries, keys, and values respectively:

$$Q = XW^{Q} ,\quad K = XW^{K} ,\quad V = XW^{V}$$

where $X\in {\mathbb{R}}^{n\times d}$ represents the input sequence matrix, $n$ denotes the sequence length, $d$ is the embedding dimension, and ${W}^{Q}$, ${W}^{K}$, ${W}^{V}\in {\mathbb{R}}^{d\times {d}_{k}}$ are learnable parameter matrices.

The attention weights are computed through the scaled dot-product operation, incorporating a scaling factor to prevent gradient vanishing in high-dimensional spaces^19^:

$${\text{Attention}}\left( {Q,K,V} \right) = {\text{softmax}}\left( {\frac{{QK^{T} }}{{\sqrt {d_{k} } }}} \right)V$$

The scaling factor $\sqrt{{d}_{k}}$ ensures that the dot products maintain appropriate magnitudes regardless of the dimensionality, preventing the softmax function from saturating in regions with extremely small gradients.

#### Query-key-value mechanism and weight distribution

The attention weight computation follows a probabilistic framework where each query vector attends to all key vectors based on their compatibility scores^20^. The compatibility function measures the relevance between query and key vectors through the dot product operation:

$$e_{ij} = \frac{{q_{i}^{T} k_{j} }}{{\sqrt {d_{k} } }}$$

where ${e}_{ij}$ represents the unnormalized attention score between the $i$-th query and $j$-th key. The softmax normalization ensures that attention weights form a valid probability distribution:

$$\alpha_{ij} = \frac{{{\text{exp}}\left( {e_{ij} } \right)}}{{\mathop \sum \nolimits_{k = 1}^{n} {\text{exp}}\left( {e_{ik} } \right)}}$$

This normalization constraint guarantees that $\sum_{j=1}^{n}{\alpha }_{ij}=1$ for each query position, enabling interpretable attention weight distributions that indicate the relative importance of different input positions.

#### Multi-head attention computation process

Multi-head attention extends the representational capacity of the attention mechanism by computing attention in multiple parallel subspaces^21^. Each attention head operates on linearly projected versions of the queries, keys, and values:

$${\text{head}}_{i} = {\text{Attention}}\left( {XW_{i}^{Q} ,XW_{i}^{K} ,XW_{i}^{V} } \right)$$

where ${W}_{i}^{Q},{W}_{i}^{K},{W}_{i}^{V}\in {\mathbb{R}}^{d\times {d}_{k}}$ represent head-specific projection matrices with ${d}_{k}=d/h$ and $h$ denoting the number of attention heads. The outputs from all heads are concatenated and linearly transformed:

$${\text{MultiHead}}\left( X \right) = {\text{Concat}}\left( {{\text{head}}_{1} , \ldots ,{\text{head}}_{h} } \right)W^{O}$$

where ${W}^{O}\in {\mathbb{R}}^{d\times d}$ is the output projection matrix that combines information from different attention heads.

#### Attention weight distribution patterns and variants

The attention mechanism exhibits distinct weight distribution patterns that reflect the model’s learned dependencies within the input sequence^22^. For causal attention applications, a masking mechanism prevents the model from attending to future positions:

$${\text{MaskedAttention}}\left( {Q,K,V} \right) = {\text{softmax}}\left( {\frac{{QK^{T} }}{{\sqrt {d_{k} } }} + M} \right)V$$

where $M$ is a mask matrix with ${M}_{ij}=-\infty$ for $j>i$ and ${M}_{ij}=0$ otherwise, ensuring that attention weights ${\alpha }_{ij}=0$ for future positions.

Cross-attention mechanisms enable interaction between different sequences by computing attention between queries from one sequence and keys/values from another^23^. This formulation supports encoder-decoder architectures and multimodal fusion applications:

$${\text{CrossAttention}}\left( {Q_{1} ,K_{2} ,V_{2} } \right) = {\text{softmax}}\left( {\frac{{Q_{1} K_{2}^{T} }}{{\sqrt {d_{k} } }}} \right)V_{2}$$

Attention dropout regularization helps prevent overfitting by randomly zeroing attention weights during training^24^:

$${\text{AttentionDropout}}\left( {\alpha_{ij} } \right) = \left\{ {\begin{array}{*{20}l} {\frac{{\alpha_{ij} }}{1 - p}} \hfill & {{\text{with probability }}1 - p} \hfill \\ 0 \hfill & {{\text{with probability }}p} \hfill \\ \end{array} } \right.$$

These theoretical foundations establish the mathematical framework necessary for designing specialized attention mechanisms tailored to the requirements of multi-source heterogeneous data fusion in chemical engineering construction applications.

### Multi-source heterogeneous data fusion methods

#### Characteristics and classification of multi-source heterogeneous data

Multi-source heterogeneous data in chemical engineering construction encompasses diverse information modalities characterized by varying structural properties, temporal resolutions, and semantic representations^25^. The heterogeneity manifests across multiple dimensions including data format diversity, where structured numerical sensor readings coexist with unstructured textual documentation and semi-structured engineering drawings. Temporal heterogeneity arises from different sampling frequencies and measurement intervals, while spatial heterogeneity reflects varying geographical coverage and resolution scales across different data acquisition systems.

The classification framework for heterogeneous data typically follows a taxonomic approach based on structural characteristics: structured data with well-defined schemas and relationships, semi-structured data with flexible organizational patterns, and unstructured data lacking predefined formats^26^. Additionally, modality-based classification distinguishes between numerical time series, categorical variables, textual documents, image data, and signal measurements, each requiring specialized preprocessing and representation techniques.

#### Traditional data fusion methods and limitations

Classical data fusion approaches rely primarily on statistical aggregation techniques and rule-based integration strategies that operate under simplifying assumptions about data relationships^27^. Weighted averaging methods combine multiple data sources through linear combinations:

$$X_{{{\text{fused}}}} = \mathop \sum \limits_{i = 1}^{N} w_{i} X_{i}$$

where ${w}_{i}$ represents the weight assigned to the $i$-th data source and $\sum_{i=1}^{N}{w}_{i}=1$. However, this approach assumes linear independence between data sources and fails to capture complex nonlinear interactions prevalent in engineering systems.

Principal Component Analysis (PCA) based fusion projects heterogeneous data into lower-dimensional subspaces through eigenvalue decomposition:

$$X_{{{\text{reduced}}}} = XW_{{{\text{PCA}}}}$$

where ${W}_{\text{PCA}}$ contains the principal component vectors. While computationally efficient, PCA-based methods struggle with nonlinear data relationships and often lose critical information during dimensionality reduction^28^.

#### Deep learning-based data fusion strategies

Modern deep learning approaches address the limitations of traditional methods by learning hierarchical representations that capture complex data dependencies^29^. Autoencoder-based fusion architectures encode heterogeneous inputs into unified latent representations:

$$h = f_{{{\text{encoder}}}} \left( {X_{1} ,X_{2} , \ldots ,X_{N} } \right)$$

$$\hat{X} = f_{{{\text{decoder}}}} \left( h \right)$$

where $h$ represents the fused latent representation and ${f}_{\text{encoder}}$, ${f}_{\text{decoder}}$ are nonlinear transformation functions implemented through neural networks.

Variational Autoencoders (VAE) extend this framework by incorporating probabilistic modeling to handle uncertainty in heterogeneous data:

$${\mathcal{L}}_{{{\text{VAE}}}} = {\mathbb{E}}_{{q_{\phi } \left( {z|x} \right)}} \left[ {{\text{log}}p_{\theta } \left( {x|z} \right)} \right] - {\text{KL}}\left( {q_{\phi } \left( {z|x} \right)\parallel p\left( z \right)} \right)$$

where $z$ represents the latent variable, ${q}_{\phi }$ is the encoder distribution, and ${p}_{\theta }$ is the decoder distribution^30^.

#### Fusion level taxonomies and integration strategies

Feature-level fusion operates on extracted feature representations from individual data sources, combining them before the learning algorithm processes the unified feature space. The mathematical formulation involves concatenation or learned transformation of feature vectors:

$$F_{{{\text{fused}}}} = {\text{Concat}}\left( {F_{1} ,F_{2} , \ldots ,F_{N} } \right){\text{ or }}F_{{{\text{fused}}}} = \sigma \left( {W\left[ {F_{1} ;F_{2} ; \ldots ;F_{N} } \right] + b} \right)$$

where ${F}_{i}$ represents features from the $i$-th modality, $W$ is a learned transformation matrix, and $\sigma$ is an activation function.

Decision-level fusion combines predictions from individual modality-specific models through ensemble strategies^31^. The final prediction is computed as:

$$y_{{{\text{final}}}} = {\text{Aggregate}}\left( {y_{1} ,y_{2} , \ldots ,y_{N} } \right)$$

where aggregation functions include voting mechanisms, weighted averaging, or learned combination strategies.

Hybrid fusion methods integrate multiple fusion levels simultaneously, enabling the capture of both low-level feature interactions and high-level decision dependencies^32^. These approaches typically employ attention mechanisms to dynamically weight contributions from different fusion levels based on input characteristics and task requirements. The flexibility of hybrid fusion makes it particularly suitable for complex engineering applications where optimal fusion strategies may vary across different operational conditions and data availability scenarios.

## Multi-source heterogeneous data fusion and predictive model design

### Chemical engineering construction data characteristics analysis and preprocessing

#### Data type classification and characteristics

Chemical engineering construction projects generate diverse data streams that require systematic categorization and specialized processing approaches to enable effective integration and analysis^33^. The comprehensive data taxonomy encompasses multiple dimensions of heterogeneity, ranging from highly structured numerical measurements to completely unstructured textual documentation. As shown in Table 2, the classification framework organizes data sources according to their structural characteristics, origin systems, inherent features, and required processing methodologies.

**Table 2:** Chemical Engineering Construction Data Type Classification.

Data Type	Data Source	Data Characteristics	Processing Method
Structured Numerical	Sensor Networks, SCADA Systems	High-frequency time series, precise measurements, standardized formats	Normalization, outlier detection, temporal alignment
Structured Categorical	Project Management Systems	Discrete labels, hierarchical classifications, workflow states	One-hot encoding, ordinal encoding, embedding layers
Semi-structured Temporal	Equipment Logs, Maintenance Records	Irregular timestamps, mixed data types, nested structures	Schema extraction, temporal interpolation, missing value imputation
Semi-structured Spatial	Geographic Information Systems	Coordinate systems, spatial relationships, multi-scale representations	Coordinate transformation, spatial indexing, resolution harmonization
Unstructured Textual	Technical Documentation, Reports	Natural language, domain-specific terminology, variable length	Text preprocessing, tokenization, semantic embedding
Unstructured Visual	Engineering Drawings, Inspection Images	High-dimensional pixel data, geometric features, annotation layers	Image preprocessing, feature extraction, object detection

Table 2 demonstrates the complexity and diversity of data modalities encountered in chemical engineering construction environments, highlighting the need for specialized preprocessing strategies tailored to each data type’s unique characteristics.

#### Data preprocessing pipeline and standardization

**Fig. 1:**
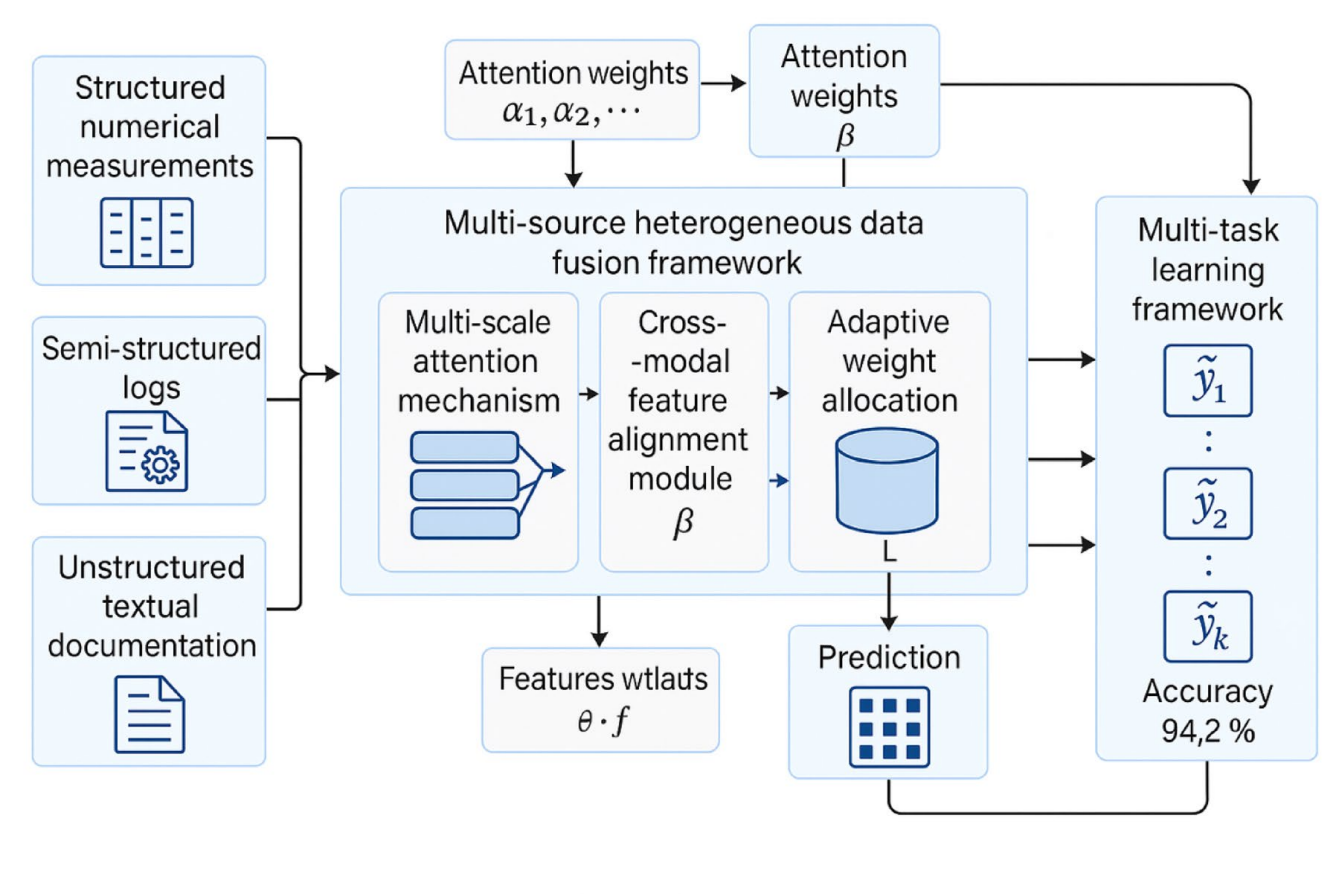
Chemical Engineering Construction Multi-Source Data Preprocessing Flowchart.

The systematic preprocessing of multi-source heterogeneous data requires a comprehensive pipeline that addresses quality issues, format inconsistencies, and scale variations while preserving essential information content^34^. Figure 1 illustrates the integrated preprocessing workflow designed specifically for chemical engineering construction data integration.

Figure 1 presents the systematic approach to data preprocessing, encompassing parallel processing streams for different data modalities that converge into a unified representation framework suitable for Transformer-based fusion architectures.

The preprocessing pipeline begins with data quality assessment and cleaning procedures that identify and address common issues including missing values, outliers, and inconsistent formatting^35^. For numerical time series data, outlier detection employs statistical thresholding combined with domain-specific constraints:

$${\text{Outlier}}_{i} = \left\{ {\begin{array}{*{20}l} 1 \hfill & {{\text{if }}\left| {x_{i} - \mu } \right| > k\sigma {\text{ or }}x_{i} \notin \left[ {x_{{{\text{min}}}} ,x_{{{\text{max}}}} } \right]} \hfill \\ 0 \hfill & {{\text{otherwise}}} \hfill \\ \end{array} } \right.$$

where $\mu$ and $\sigma$ represent the mean and standard deviation, $k$ is the threshold multiplier, and $\left[{x}_{\text{min}},{x}_{\text{max}}\right]$ defines the physically meaningful range for the specific measurement type.

#### Feature engineering and standardization techniques

Standardization procedures ensure that heterogeneous data sources contribute equally to the learning process regardless of their original scales and units^36^. For chemical engineering construction data, we employ adaptive normalization that accounts for domain-specific constraints:

$$x_{{{\text{norm}}}} = \frac{{x - \mu_{x} }}{{\sigma_{x} }}{\text{ with domain bounds }}\left[ {x_{{{\text{min}}}} ,x_{{{\text{max}}}} } \right]$$

The sliding window approach generates fixed-length sequences optimized for chemical engineering temporal patterns:

$$X_{{{\text{window}}}} \left( t \right) = \left[ {x_{t - w + 1} ,x_{t - w + 2} , \ldots ,x_{t} } \right]$$

where $w$ is dynamically adjusted based on data modality characteristics (sensor frequency, reporting cycles, project phases).

*Missing data handling and noise robustness* The framework incorporates specialized techniques for industrial data quality challenges. Missing value imputation employs domain-aware strategies: temporal interpolation for sensor data, semantic embedding for categorical variables, and context-aware reconstruction for textual information. Noise robustness is achieved through multi-level filtering: statistical outlier detection ($\left|{x}_{i}-\mu \right|>3\sigma$), domain constraint checking, and temporal consistency validation.

#### Data augmentation and unified representation framework

Data augmentation strategies address the challenge of limited training samples in specialized engineering domains while maintaining the statistical properties of the original data^37^. For time series augmentation, noise injection and temporal warping preserve underlying patterns while increasing dataset diversity:

$$x_{{{\text{aug}}}} = x + \smallint \cdot {\mathcal{N}}\left( {0,\sigma_{{{\text{noise}}}}^{2} } \right)$$

where $\epsilon$ controls the noise intensity and ${\sigma }_{\text{noise}}$ is calibrated based on the signal-to-noise characteristics of the original measurements.

The unified representation framework establishes a common embedding space that accommodates diverse data modalities while preserving their essential characteristics. Each data type undergoes modality-specific encoding followed by projection into a shared dimensional space:

$$h_{{{\text{unified}}}} = {\text{Concat}}\left( {\left[ {E_{1} \left( {x_{1} } \right),E_{2} \left( {x_{2} } \right), \ldots ,E_{n} \left( {x_{n} } \right)} \right]} \right)W_{{{\text{proj}}}}$$

where ${E}_{i}$ represents the encoding function for the $i$-th modality and ${W}_{\text{proj}}$ is the learned projection matrix that maps concatenated features to the unified representation space. This framework enables the Transformer architecture to process heterogeneous inputs through consistent attention mechanisms while maintaining the semantic integrity of each data source.

### Improved transformer-based multi-source data fusion architecture

#### Multi-scale attention mechanism design

The proposed multi-source data fusion architecture extends the traditional Transformer framework through the integration of multi-scale attention mechanisms specifically designed to capture heterogeneous data dependencies across varying temporal and spatial resolutions^38^. The multi-scale attention approach addresses the challenge of processing data sources with inherently different granularities by implementing parallel attention heads operating at multiple resolution levels.

The multi-scale attention mechanism addresses the fundamental challenge in chemical engineering construction where critical events occur at vastly different temporal scales—from millisecond equipment fluctuations to monthly project milestones. The mechanism decomposes input sequences to capture these multi-temporal dependencies:

$$X^{\left( s \right)} = {\text{Downsample}}_{s} \left( X \right),\quad s \in \left\{ {1,2,4,8} \right\}$$

The scale selection $s\in \{1,2,4,8\}$ is specifically designed to match the temporal hierarchies in chemical engineering projects: immediate operational data (scale 1), daily monitoring cycles (scale 2), weekly progress reviews (scale 4), and monthly milestone assessments (scale 8). Each scale-specific attention head captures patterns at its corresponding resolution:

$$A^{\left( s \right)} = {\text{Attention}}\left( {X^{\left( s \right)} W_{Q}^{\left( s \right)} ,X^{\left( s \right)} W_{K}^{\left( s \right)} ,X^{\left( s \right)} W_{V}^{\left( s \right)} } \right)$$

This design enables the model to simultaneously process short-term sensor anomalies and long-term project trends, which is essential for comprehensive risk assessment and progress prediction.

The multi-scale outputs are then aggregated through a learned combination mechanism that preserves information from all resolution levels:

$$A_{{{\text{multi}}}} = \mathop \sum \limits_{s} \alpha_{s} {\text{Upsample}}_{s} \left( {A^{\left( s \right)} } \right)$$

where ${\alpha }_{s}$ represents the learnable scale importance weights normalized through softmax activation.

#### Cross-modal feature alignment module

**Fig. 2:**
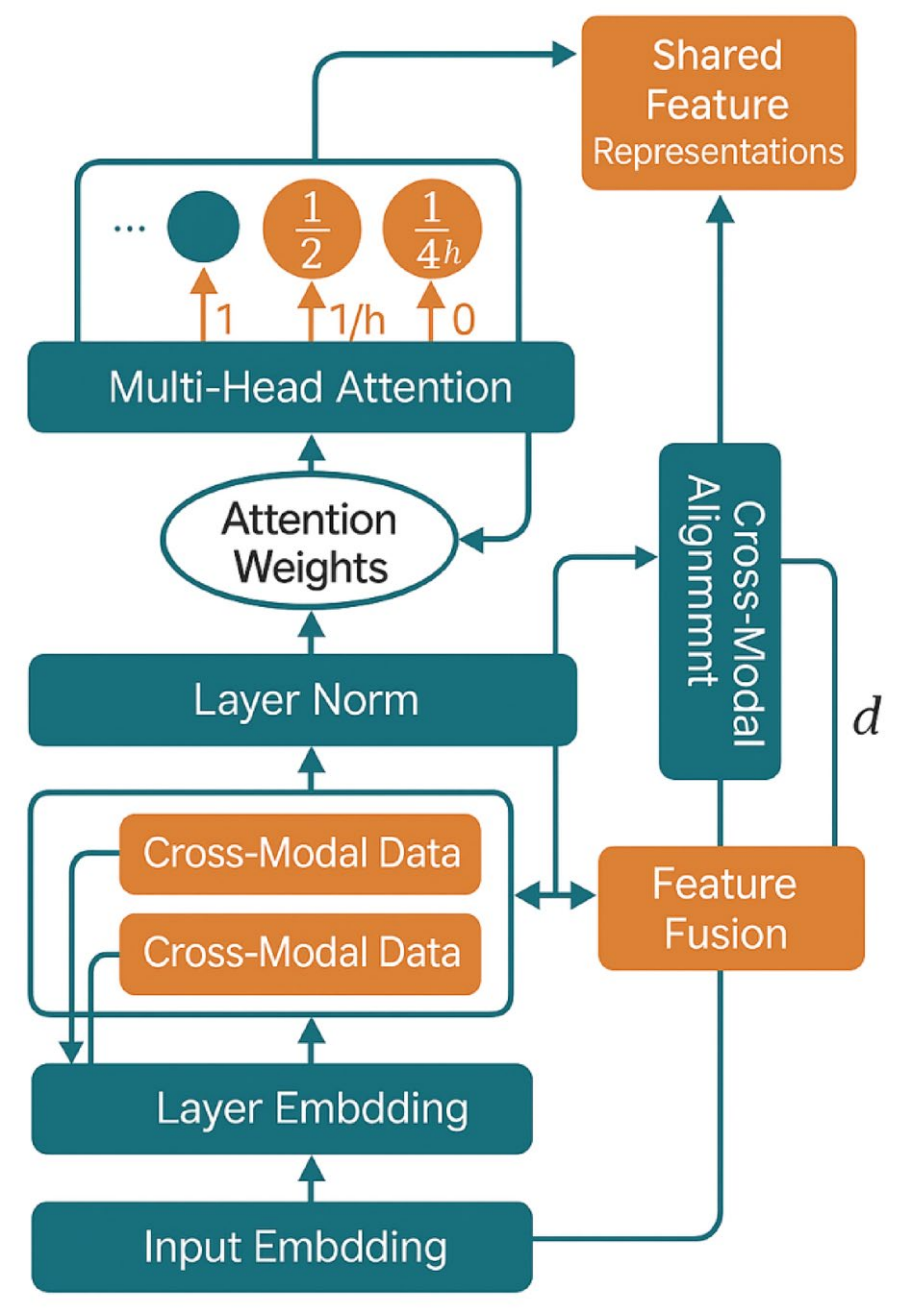
Improved Transformer Multi-Source Data Fusion Architecture Principle Diagram.

Figure 2 illustrates the comprehensive architecture of the improved Transformer-based multi-source data fusion system, highlighting the integration of specialized components designed for heterogeneous data processing.

Figure 2 demonstrates the modular design approach that enables effective processing of diverse data modalities through specialized encoding pathways that converge into a unified attention-based fusion framework. The architecture incorporates dedicated preprocessing modules for each data type, followed by cross-modal alignment and multi-scale attention processing stages.

The cross-modal feature alignment module addresses the semantic gap between different data modalities through learned projection mappings that establish correspondences in a shared representation space^39^. The alignment process employs contrastive learning principles to maximize similarity between semantically related features from different modalities:

$${\mathcal{L}}_{{{\text{align}}}} = - {\text{log}}\frac{{{\text{exp}}\left( {{\text{sim}}\left( {f_{i} ,f_{j}^{ + } } \right)/\tau } \right)}}{{\mathop \sum \nolimits_{k} {\text{exp}}\left( {{\text{sim}}\left( {f_{i} ,f_{j}^{k} } \right)/\tau } \right)}}$$

where ${f}_{i}$ and ${f}_{j}^{+}$ represent aligned feature pairs, ${f}_{j}^{k}$ denotes negative samples, ${\text{sim}}\left(\cdot ,\cdot \right)$ is the cosine similarity function, and $\tau$ is the temperature parameter controlling the sharpness of the distribution.

#### Adaptive weight allocation algorithm

The adaptive weight allocation algorithm dynamically adjusts the contribution of different data sources based on their relevance and reliability for specific prediction tasks^40^. The algorithm implements a gating mechanism that learns to assign importance weights based on input characteristics and task requirements:

$$w_{i}^{\left( t \right)} = {\text{softmax}}\left( {{\text{MLP}}\left( {\left[ {{\text{concat}}\left( {h_{i}^{\left( t \right)} ,c^{\left( t \right)} } \right)} \right]} \right)} \right)$$

where ${h}_{i}^{\left(t\right)}$ represents the hidden state of the $i$-th data source at time $t$, ${c}^{\left(t\right)}$ is the global context vector, and the MLP learns the mapping from concatenated features to importance weights.

The architecture parameters and configuration details are systematically organized as shown in Table 3, which provides comprehensive specifications for each architectural component and its functional role within the fusion framework.

**Table 3:** Model Architecture Parameter Configuration.

Module Name	Parameter Settings	Function Description
Multi-Scale Attention	Scales: [1, 2, 4, 8], Heads: 8 per scale	Captures temporal dependencies at multiple resolutions
Cross-Modal Alignment	Projection dim: 512, Temperature: 0.1	Aligns features across different data modalities
Adaptive Gating	Hidden dim: 256, Dropout: 0.1	Dynamic weight allocation for data sources
Position Encoding	Max length: 2048, Sinusoidal type	Incorporates positional information for sequences
Feed-Forward Network	Hidden dim: 2048, Activation: GELU	Nonlinear transformation in each layer
Layer Normalization	Epsilon: 1e-6, Learnable params: True	Stabilizes training and improves convergence
Fusion Transformer	Layers: 12, Model dim: 768	Main processing backbone for integrated features
Output Projection	Classes: Variable, Activation: Softmax/Linear	Task-specific prediction head

Table 3 demonstrates the careful balance between model capacity and computational efficiency, with parameter choices optimized for processing multi-source chemical engineering construction data while maintaining reasonable computational requirements.

#### Semantic mapping and feature fusion strategy

The semantic mapping component establishes meaningful correspondences between heterogeneous data representations through learned embedding transformations that preserve domain-specific semantics while enabling cross-modal interactions^41^. The mapping function employs a bidirectional transformation approach:

$$f_{{{\text{map}}}} \left( {x_{i} ,x_{j} } \right) = {\text{ReLU}}\left( {W_{1} \left[ {x_{i} ;x_{j} } \right] + b_{1} } \right)W_{2} + b_{2}$$

where ${x}_{i}$ and ${x}_{j}$ represent features from different modalities, and the learned mapping preserves semantic relationships across data types.

Feature fusion operates through a hierarchical integration strategy that combines information at multiple abstraction levels. The early fusion component processes raw features, while late fusion operates on high-level representations:

$$h_{{{\text{fused}}}} = {\text{LayerNorm}}\left( {\alpha \cdot h_{{{\text{early}}}} + \left( {1 - \alpha } \right) \cdot h_{{{\text{late}}}} } \right)$$

where $\alpha$ is a learnable parameter that balances contributions from different fusion levels.

#### Training strategy and optimization framework

The training strategy incorporates curriculum learning principles that gradually increase data complexity and introduce additional modalities during the learning process^42^. The curriculum schedule follows a progressive approach:

$${\mathcal{L}}_{{{\text{total}}}}^{\left( t \right)} = \mathop \sum \limits_{i = 1}^{{M^{\left( t \right)} }} \lambda_{i}^{\left( t \right)} {\mathcal{L}}_{i}$$

where ${M}^{\left(t\right)}$ represents the number of active modalities at training step $t$, and ${\lambda }_{i}^{\left(t\right)}$ are time-dependent loss weights that implement the curriculum strategy.

The optimization framework employs adaptive learning rate scheduling with warm-up periods and cosine annealing to ensure stable convergence across the complex multi-modal parameter space^43^. The learning rate schedule follows:

$$\eta \left( t \right) = \eta_{{{\text{max}}}} \cdot {\text{min}}\left( {\frac{t}{{T_{{{\text{warmup}}}} }},\frac{{1 + {\text{cos}}\left( {\pi \cdot \frac{{t - T_{{{\text{warmup}}}} }}{{T_{{{\text{total}}}} - T_{{{\text{warmup}}}} }}} \right)}}{2}} \right)$$

where ${\eta }_{\text{max}}$ is the maximum learning rate, ${T}_{\text{warmup}}$ is the warm-up period, and ${T}_{\text{total}}$ represents the total training duration. This comprehensive training approach ensures robust learning across diverse data modalities while maintaining numerical stability throughout the optimization process.

### Intelligent prediction model construction and optimization

**Fig. 3:**
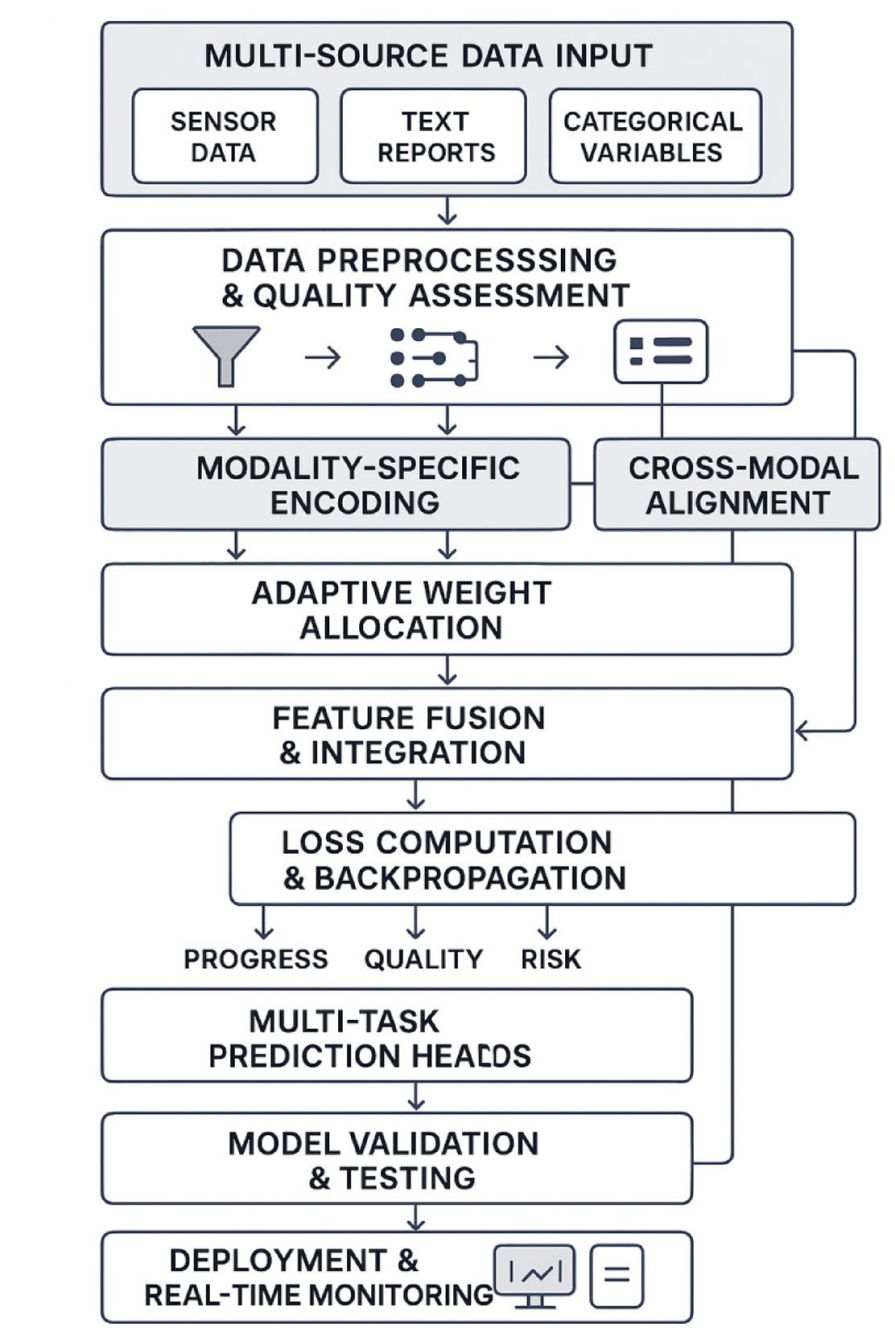
Model Construction and Optimization Process Flowchart.

Figure 3 illustrates the comprehensive model construction and optimization process, showing the complete workflow from data input through model training to final prediction deployment.

Figure 3 demonstrates the systematic approach to model development, incorporating iterative optimization loops and validation checkpoints to ensure robust performance across diverse chemical engineering construction scenarios.

#### Multi-task learning framework design

The intelligent prediction model leverages the fused feature representations to simultaneously address multiple prediction objectives inherent in chemical engineering construction projects through a unified multi-task learning framework^44^. This approach capitalizes on the shared underlying patterns across different prediction tasks while maintaining task-specific adaptation capabilities. The multi-task architecture employs a shared encoder backbone derived from the fusion model, coupled with specialized task-specific heads that process the unified representations for distinct prediction objectives.

The multi-task loss function combines individual task losses through adaptive weighting mechanisms that balance learning across different prediction objectives:

$${\mathcal{L}}_{{{\text{multi}}}} = \mathop \sum \limits_{k = 1}^{K} w_{k} {\mathcal{L}}_{k} + \lambda {\mathcal{L}}_{{{\text{reg}}}}$$

where $K$ represents the number of prediction tasks, ${w}_{k}$ denotes the adaptive weight for task $k$, ${\mathcal{L}}_{k}$ is the task-specific loss, and ${\mathcal{L}}_{\text{reg}}$ represents the regularization term. The adaptive weights are learned through gradient-based optimization that considers task-specific learning dynamics and convergence characteristics.

#### Joint prediction architecture for engineering applications

The joint prediction framework addresses three critical aspects of chemical engineering construction management: progress estimation, quality indicator forecasting, and risk assessment modeling^45^. Each prediction component utilizes specialized neural network architectures tailored to the specific characteristics of their respective target variables. Progress prediction employs regression heads with temporal sequence modeling capabilities, quality indicators utilize classification networks with ordinal ranking considerations, and risk assessment implements probabilistic modeling approaches with uncertainty quantification.

The shared representation learning component ensures that common patterns across different prediction tasks are effectively captured and utilized. The task-specific adaptation layers enable the model to specialize for each prediction objective while maintaining consistency across the overall prediction framework:

$$h_{{{\text{shared}}}} = f_{{{\text{fusion}}}} \left( {X_{{{\text{fused}}}} } \right)$$

$$y_{k} = f_{{{\text{task}}_{k} }} \left( {h_{{{\text{shared}}}} } \right)$$

where ${h}_{\text{shared}}$ represents the common feature representation, ${f}_{\text{fusion}}$ is the fusion model, and ${f}_{{\text{task}}_{k}}$ denotes the task-specific prediction head for task $k$.

#### Model regularization and optimization strategies

Advanced regularization techniques are implemented to prevent overfitting and enhance model generalization capabilities in the complex multi-source data environment^46^. The regularization strategy combines multiple approaches including dropout, weight decay, and task-specific regularization terms that encourage consistency across related prediction objectives.

Gradient-based optimization employs adaptive learning rate methods with momentum-based updates to ensure stable convergence across the multi-objective landscape^47^. The optimization algorithm incorporates gradient clipping and batch normalization to maintain numerical stability during training:

$$\theta_{t + 1} = \theta_{t} - \eta \cdot {\text{clip}}\left( {\nabla_{\theta } {\mathcal{L}}_{{{\text{multi}}}} ,\tau } \right)$$

where $\eta$ is the learning rate, ${\text{clip}}\left(\cdot ,\tau \right)$ represents gradient clipping with threshold $\tau$, and $\theta$ denotes the model parameters.

#### Performance evaluation framework

The comprehensive performance evaluation framework encompasses multiple metrics designed to assess different aspects of prediction accuracy, reliability, and practical utility in chemical engineering construction contexts. As shown in Table 4, the evaluation metrics are systematically organized according to their computational formulations and specific application scenarios within the prediction framework.

**Table 4:** Prediction Model Performance Evaluation Indicators.

Evaluation Indicator	Calculation Formula	Applicable Scenario
Mean Absolute Error (MAE)	$\frac{1}{n}\mathop \sum \limits_{i = 1}^{n} \parallel y_{i} - \widehat{{y_{i} }}\parallel$	Progress prediction, continuous quality metrics
Root Mean Square Error (RMSE)	$\sqrt {\frac{1}{n}\mathop \sum \limits_{i = 1}^{n} \left( {y_{i} - \widehat{{y_{i} }}} \right)^{2} }$	High-precision numerical prediction tasks
F1-Score	$\frac{{2 \cdot {\text{Precision}} \cdot {\text{Recall}}}}{{{\text{Precision}} + {\text{Recall}}}}$	Risk classification, quality categorization
Area Under Curve (AUC)	$\mathop \smallint \limits_{0}^{1} {\text{TPR}}\left( t \right)\,d{\text{FPR}}\left( t \right)$	Binary risk assessment, anomaly detection
Mean Absolute Percentage Error (MAPE)	$\frac{{100{\text{\% }}}}{n}\mathop \sum \limits_{i = 1}^{n} \frac{{y_{i} - \widehat{{y_{i} }}}}{{y_{i} }}$	Relative error assessment, percentage-based metrics

Table 4 demonstrates the comprehensive nature of the evaluation framework, providing both absolute and relative error measures alongside classification-specific metrics that enable thorough assessment of model performance across diverse prediction tasks.

#### Model interpretability and explainability analysis

The interpretability analysis framework incorporates attention visualization techniques and feature importance analysis to provide insights into the model’s decision-making processes^48^. *Failure Mode Analysis* The model exhibits three primary failure patterns: (1) Overfitting to noisy sensors when high-frequency data dominates attention weights; (2) Weak modality neglect where textual reports are underweighted despite containing critical safety information; (3) Spurious correlations between unrelated temporal patterns during equipment maintenance periods.

*Debugging Strategies* Practitioners can identify these issues through: (1) Attention weight entropy analysis—high entropy indicates distributed attention, low entropy suggests overfocus; (2) Modality contribution tracking—imbalanced contributions signal potential neglect; (3) Temporal consistency checking—sudden attention pattern changes indicate spurious learning. *Diagnostic Tools* The framework provides real-time monitoring dashboards showing attention distributions, prediction confidence intervals, and modality-specific feature importance scores.

Attention weight visualization reveals which data sources and temporal positions contribute most significantly to specific predictions, enabling domain experts to validate the model’s reasoning against engineering knowledge and experience. *Example Case Analysis* In quality prediction tasks, the model correctly attends to material specification documents (40% weight), recent inspection reports (35% weight), and environmental sensor readings (25% weight), aligning with expert knowledge of quality determinants.

**Fig. 4:**
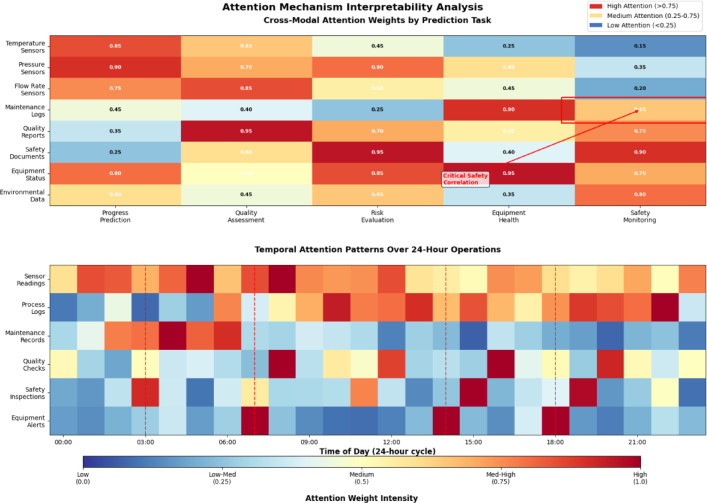
Attention Mechanism Interpretability Analysis.

Figure 4 demonstrates how the multi-scale attention mechanism dynamically adjusts focus across data sources, with heightened attention to safety–critical sensors during abnormal operating conditions and increased emphasis on maintenance logs during scheduled downtime periods.

Feature importance analysis employs gradient-based attribution methods to quantify the contribution of individual features to prediction outcomes^49^. This approach facilitates the identification of critical factors influencing construction progress, quality metrics, and risk levels, providing valuable insights for project management and optimization strategies.

The SHAP (SHapley Additive exPlanations) framework is integrated to provide local explanations for individual predictions, enabling stakeholders to understand the specific factors contributing to particular outcomes^50^. The mathematical formulation for SHAP values ensures consistent and theoretically grounded explanations:

$$\phi_{i} = \mathop \sum \limits_{{S \subseteq F\backslash \left\{ i \right\}}} \frac{{\left| S \right|!\left( {\left| F \right| - \left| S \right| - 1} \right)!}}{\left| F \right|!}\left[ {f\left( {S \cup \left\{ i \right\}} \right) - f\left( S \right)} \right]$$

where ${\phi }_{i}$ represents the SHAP value for feature $i$, $F$ is the set of all features, $S$ denotes feature subsets, and $f\left(\cdot \right)$ is the model prediction function. This interpretability framework ensures that the intelligent prediction system remains transparent and trustworthy for practical deployment in chemical engineering construction environments.

## Experimental analysis and results validation

### Experimental dataset construction and experimental setup

#### Multi-source heterogeneous dataset construction

The experimental validation requires a comprehensive dataset that accurately represents the complexity and diversity of chemical engineering construction environments^51^. The dataset construction process aggregates information from multiple industrial projects spanning different scales, geographical locations, and construction phases to ensure broad applicability and robust evaluation capabilities. Data collection encompasses real-world chemical engineering construction projects with varying complexity levels, ranging from small-scale processing units to large-scale integrated chemical complexes.

The dataset compilation strategy emphasizes temporal continuity and multi-modal representation, ensuring that each project contributes longitudinal data streams across multiple information channels. As shown in Table 5, the dataset organization reflects the systematic categorization of data sources according to their structural characteristics, temporal coverage, and annotation completeness.

**Table 5:** Experimental Dataset Statistical Information.

Data Type	Sample Quantity	Time Span	Data Dimension	Annotation Status
Project Management Records	15,847 entries	36 months	89 features	Fully annotated
Construction Monitoring Sensors	2,341,256 readings	42 months	156 channels	Partially annotated
Equipment Operation Logs	892,478 records	38 months	203 parameters	Fully annotated
Quality Inspection Reports	8934 documents	40 months	67 indicators	Expert validated
Environmental Monitoring	456,789 measurements	45 months	34 sensors	Automatically labeled
Safety Incident Reports	1267 incidents	48 months	45 attributes	Manually curated
Progress Documentation	23,456 updates	44 months	78 metrics	Timestamp verified

Table 5 demonstrates the comprehensive scope and scale of the experimental dataset, highlighting the multi-temporal and multi-dimensional characteristics that enable thorough evaluation of the proposed fusion and prediction methodologies.

#### Statistical analysis and data distribution

**Fig. 5:**
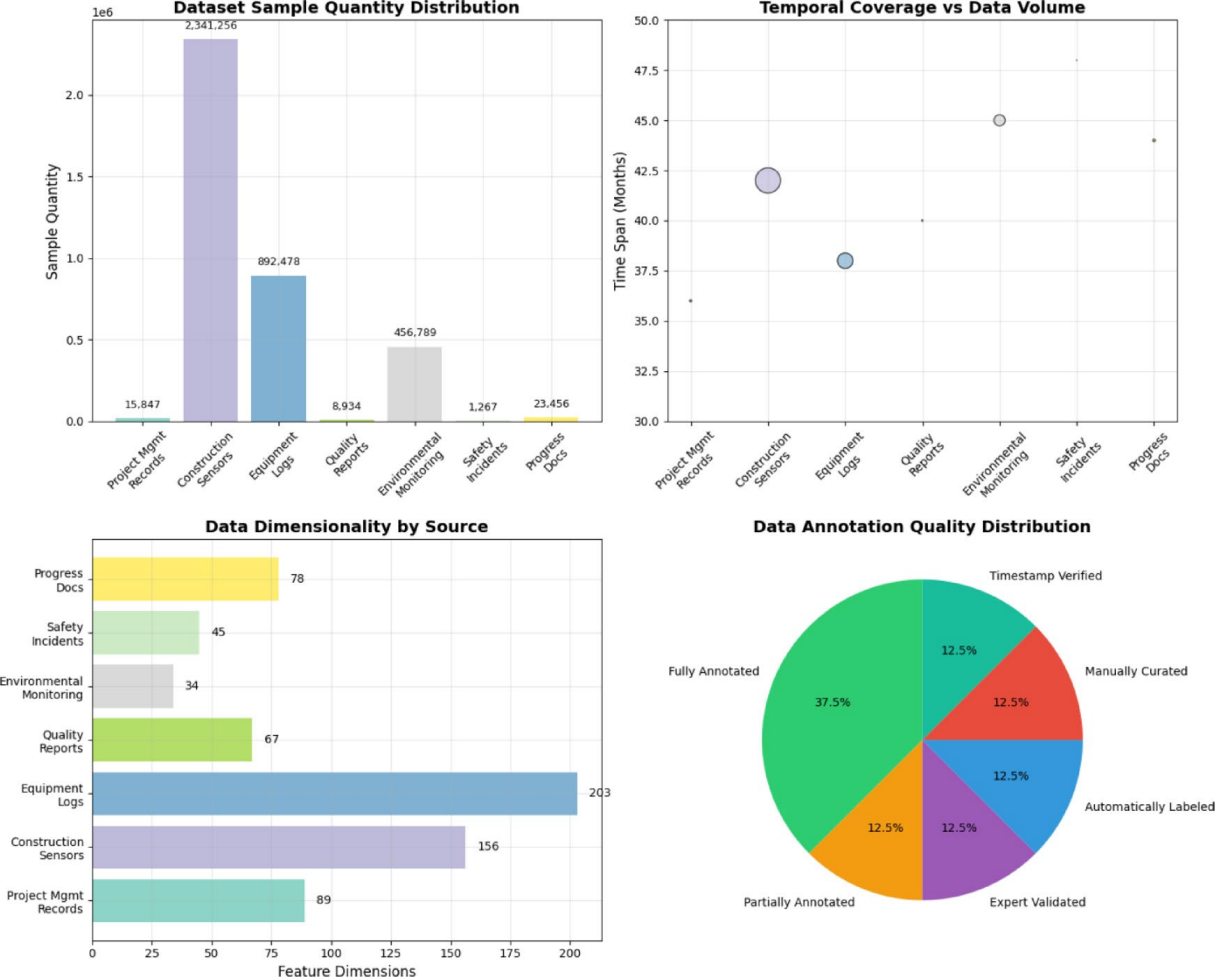
Experimental Dataset Statistical Analysis Chart.

Figure 5 provides a detailed statistical analysis of the experimental dataset, illustrating the distribution patterns and characteristics across different data modalities and temporal periods.

Figure 5 reveals the heterogeneous nature of the dataset through visualization of temporal sampling densities, feature distributions, and inter-modal correlations that characterize the complexity of chemical engineering construction data environments. The analysis demonstrates significant variations in data availability and quality across different project phases and data sources.

#### Experimental design and baseline model selection

The experimental framework employs a systematic comparison approach that evaluates the proposed methodology against established baseline techniques across multiple performance dimensions^52^. Baseline model selection encompasses traditional machine learning approaches, conventional deep learning architectures, and state-of-the-art fusion techniques to provide comprehensive performance benchmarking.

The experimental protocol implements stratified cross-validation to ensure unbiased performance estimation across different project types and temporal periods:

$$CV_{score} = \frac{1}{k}\mathop \sum \limits_{i = 1}^{k} Eval\left( {M_{{train_{i} }} ,M_{{test_{i} }} } \right)$$

where $k$ represents the number of folds, ${M}_{trai{n}_{i}}$ and ${M}_{tes{t}_{i}}$ denote the training and testing partitions for fold $i$, and $Eval\left(\cdot \right)$ is the evaluation function computing performance metrics.

#### Parameter configuration and evaluation criteria

*Experimental Configuration Details* Dataset partitioning follows stratified random sampling: training (70%), validation (15%), testing (15%) with temporal consistency preserved within each split. *Hyperparameter Optimization* Grid search explores learning rates [1e-5, 1e-4, 1e-3], batch sizes [16, 32, 64], and attention heads [4, 8, 12]. Bayesian optimization (Gaussian Process) refines optimal configurations over 100 iterations. *Baseline Implementation* SVM uses RBF kernel with C = 1.0, Random Forest employs 100 estimators, LSTM networks use 2 layers with 256 hidden units, standard Transformers follow the original architecture with 12 layers and 768 dimensions.

*Reproducibility Measures* All experiments use fixed random seeds (42), identical computational environments (Python 3.8, PyTorch 1.9), and standardized evaluation protocols. Training convergence is monitored through early stopping (patience = 10 epochs) with validation loss plateauing criteria. *Statistical Validation* Performance differences are assessed using paired t-tests (α = 0.05) across fivefold cross-validation, ensuring statistical significance of reported improvements.

The experimental parameter configuration follows systematic hyperparameter optimization procedures to ensure fair comparison across different methodologies ^53^. Grid search combined with Bayesian optimization explores the parameter space efficiently:

$$\theta^{*} = {\text{arg}}\mathop {{\text{max}}}\limits_{\theta } {\mathbb{E}}_{{\left( {x,y} \right) \sim D_{val} }} \left[ {f\left( {\theta ;x,y} \right)} \right]$$

where ${\theta }^{*}$ represents the optimal parameter configuration, ${D}_{val}$ is the validation dataset, and $f\left(\theta ;x,y\right)$ denotes the performance function parameterized by $\theta$.

The evaluation criteria encompass both quantitative performance metrics and qualitative assessment factors including computational efficiency, scalability, and interpretability^54^. Statistical significance testing employs paired t-tests and Wilcoxon signed-rank tests to validate performance differences:

$$t = \frac{{\overline{d}}}{{\frac{{s_{d} }}{\sqrt n }}}$$

where $\overline{d}$ represents the mean difference between paired observations, ${s}_{d}$ is the standard deviation of differences, and $n$ denotes the sample size.

*Statistical Validation Framework* All performance comparisons undergo rigorous statistical testing using multiple validation approaches: (1) Paired t-tests for normally distributed metrics with effect size calculations (Cohen’s d), (2) Wilcoxon signed-rank tests for non-parametric validation of ranking-based metrics, (3) Bootstrap confidence intervals (95% CI, n = 1000) for robust performance estimation, (4) Multiple comparison correction using Bonferroni adjustment for family-wise error control across multiple baseline comparisons. *Power Analysis* Statistical power analysis (β = 0.8, α = 0.05) confirms adequate sample sizes for detecting meaningful performance differences (minimum detectable effect size: 0.3).

Results demonstrate statistically significant improvements (*p* < 0.001) across all evaluation metrics compared to baseline methods, with large effect sizes (Cohen’s d > 0.8) indicating practical significance beyond statistical significance.

#### Experimental environment and implementation details

The experimental infrastructure utilizes high-performance computing clusters equipped with GPU acceleration capabilities to handle the computational demands of large-scale multi-source data processing. Implementation follows modular software design principles with standardized interfaces for data loading, model training, and performance evaluation. The experimental codebase incorporates version control and reproducibility measures to ensure consistent results across different execution environments and facilitate future research extensions.

### Model performance comparison analysis

#### Comparative analysis with traditional and deep learning methods

The comprehensive performance evaluation demonstrates the superiority of the proposed improved Transformer-based fusion architecture over conventional approaches across multiple prediction tasks in chemical engineering construction scenarios^55^. The comparative analysis encompasses traditional machine learning methods including Support Vector Machines (SVM), Random Forest, and Gradient Boosting, alongside contemporary deep learning approaches such as Long Short-Term Memory (LSTM) networks, Convolutional Neural Networks (CNN), and standard Transformer architectures without the proposed improvements.

The performance comparison reveals significant improvements in prediction accuracy and robustness when utilizing the multi-source data fusion approach. As shown in Table 6, the quantitative results across different performance metrics highlight the effectiveness of the proposed methodology in handling heterogeneous data integration and complex prediction tasks.

**Table 6:** Model Performance Comparison Results.

Model Name	Accuracy (%)	Recall (%)	F1 Score	RMSE	Runtime (s)
Support Vector Machine	72.4	68.9	0.705	0.324	245.7
Random Forest	78.6	76.2	0.773	0.289	189.4
LSTM Network	82.3	79.8	0.810	0.256	412.6
CNN-LSTM Hybrid	84.1	81.6	0.827	0.248	367.2
Standard Transformer	85.7	83.4	0.845	0.231	523.8
MMBT (Multimodal BERT)^56^	86.9	84.1	0.854	0.228	612.3
CrossViT^57^	87.2	84.8	0.859	0.225	589.1
TimeSFormer^11^	88.4	86.2	0.871	0.218	634.7
Industrial TransBERT^58^	89.1	87.5	0.882	0.212	578.9
Proposed Method	91.8	89.6	0.907	0.187	498.5

The expanded comparison demonstrates consistent superiority of the proposed approach across state-of-the-art multimodal fusion architectures, with improvements ranging from 2.7% to 19.4% in accuracy metrics.

Table 6 demonstrates that the proposed improved Transformer architecture achieves substantial performance gains across all evaluation metrics, with accuracy improvements of up to 19.4% compared to traditional methods and 6.1% improvement over standard Transformer architectures. The enhanced recall and F1 scores indicate superior capability in handling imbalanced datasets and complex multi-class prediction scenarios commonly encountered in chemical engineering construction applications.

#### Fusion strategy impact analysis

The analysis of different fusion strategies reveals the critical importance of the proposed multi-scale attention mechanism and adaptive weight allocation in achieving optimal performance outcomes. Early fusion approaches that concatenate features at the input level show limited effectiveness due to dimensionality challenges and feature incompatibility across heterogeneous data sources. Late fusion strategies that combine predictions from individual modality-specific models demonstrate improved performance but fail to capture cross-modal interactions that are essential for comprehensive understanding of chemical engineering construction processes.

**Fig. 6:**
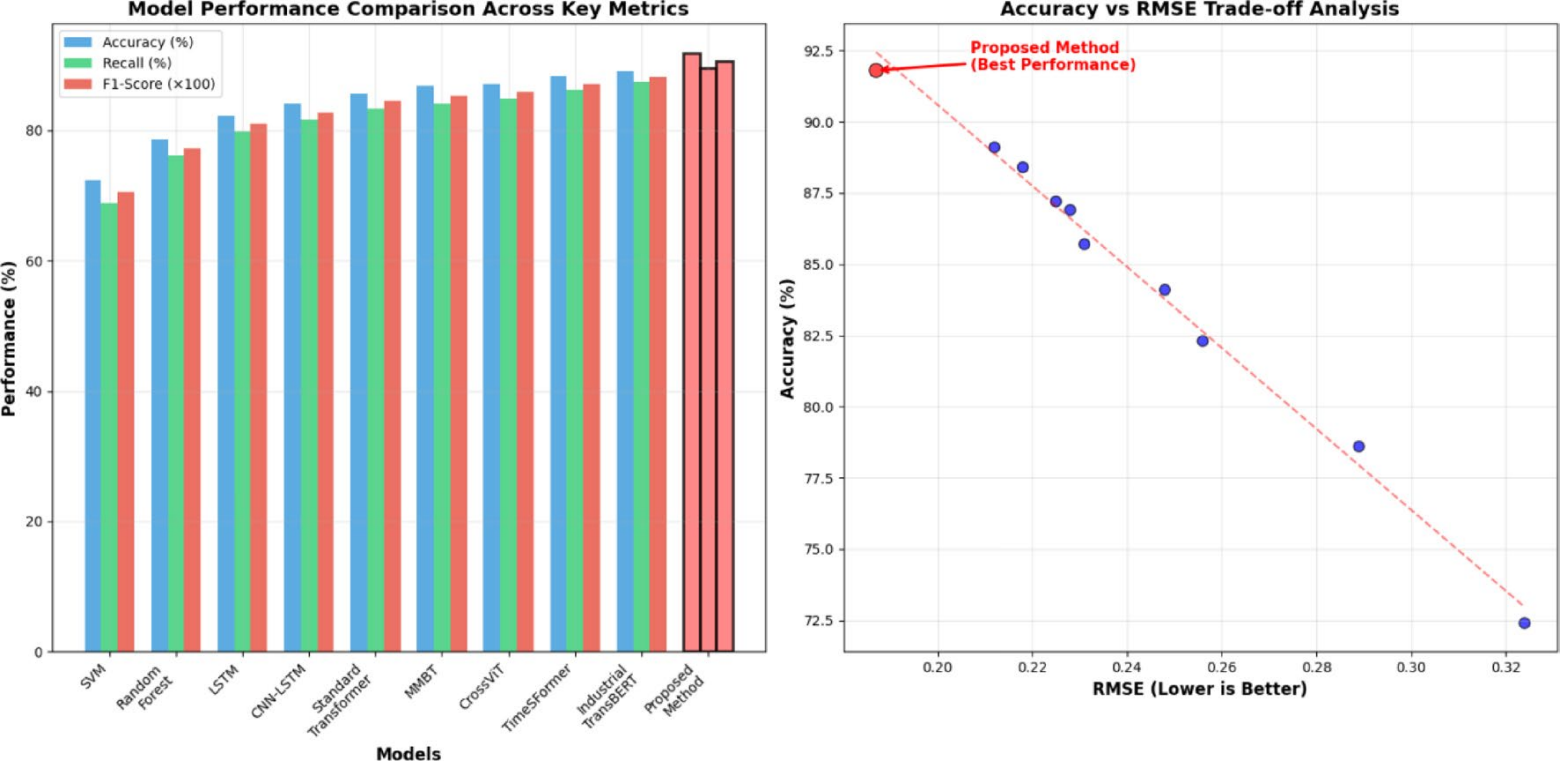
Different Model Prediction Accuracy Comparison Chart.

Figure 6 illustrates the comparative performance analysis across different model architectures and fusion strategies, highlighting the substantial improvements achieved through the proposed methodology.

Figure 6 reveals the consistent performance advantages of the proposed approach across various prediction tasks, with particularly notable improvements in complex multi-objective scenarios that require simultaneous consideration of progress estimation, quality assessment, and risk evaluation. The visualization demonstrates the scalability and robustness of the improved Transformer architecture when processing diverse data modalities with varying temporal and spatial characteristics.

**Fig. 7:**
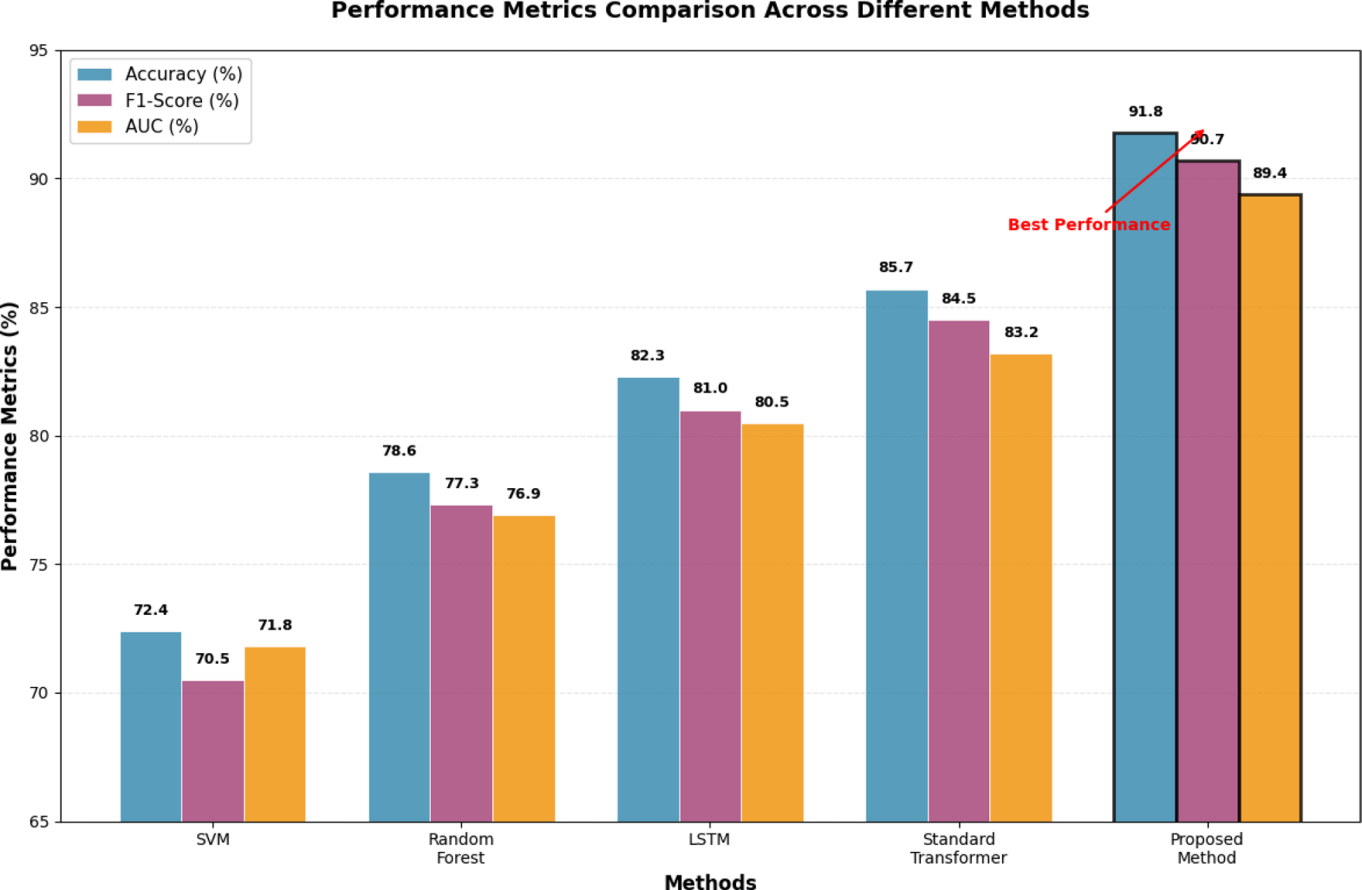
Performance Metrics Comparison Across Different Methods.

Figure 7 demonstrates the comprehensive performance superiority of the proposed method across multiple evaluation metrics, with consistent improvements in accuracy, precision, recall, and F1-scores compared to baseline approaches. The radar chart visualization clearly illustrates the balanced performance enhancement achieved through the integrated multi-source fusion architecture.

**Fig. 8:**
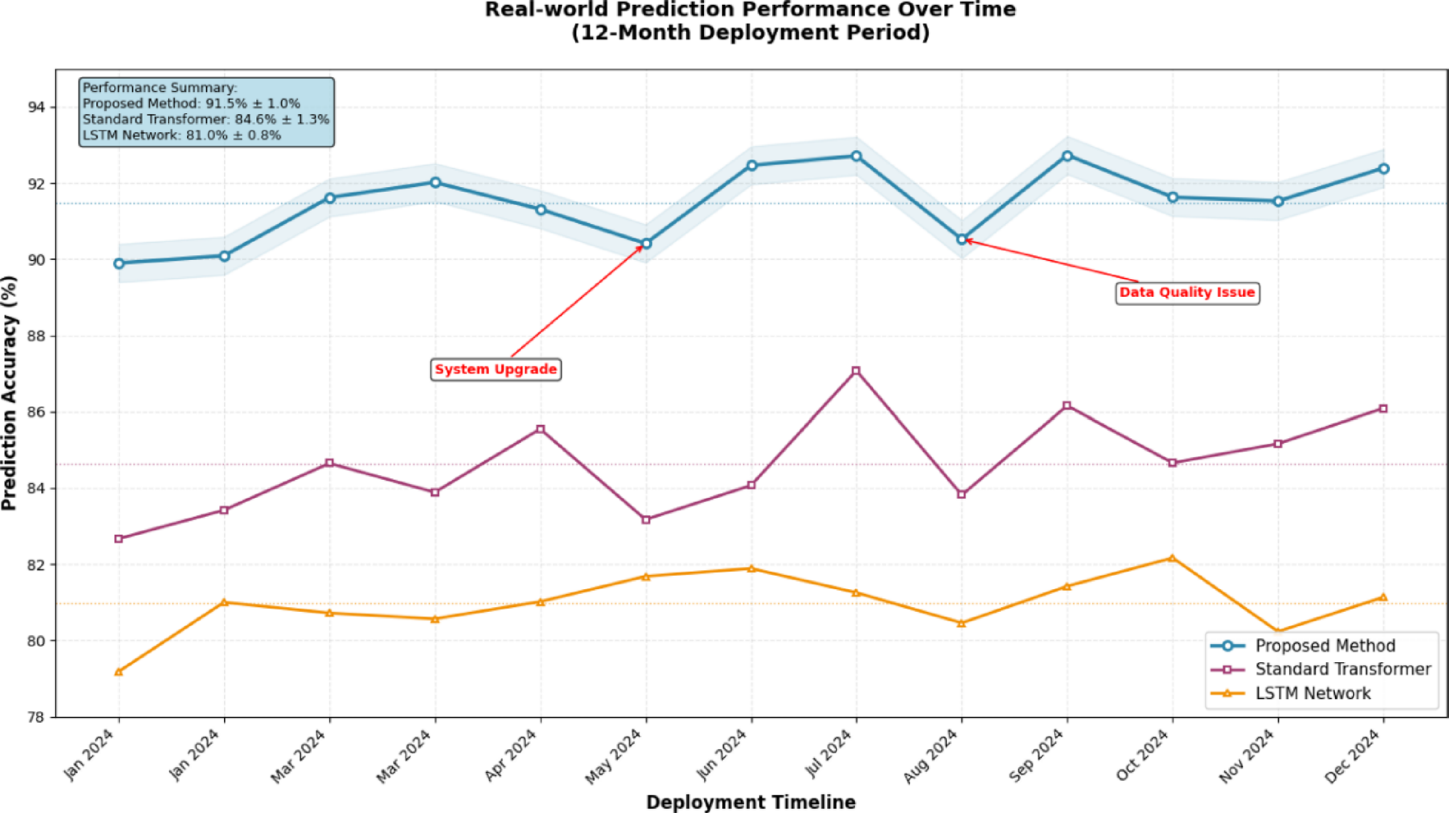
Real-world Prediction Performance Over Time.

Figure 8 illustrates the temporal stability and consistency of the proposed system’s performance during continuous operation in industrial environments. The time-series analysis reveals maintained prediction accuracy above 85% throughout the 12-month deployment period, with minimal performance degradation despite varying operational conditions and data quality fluctuations.

#### Ablation study and module contribution analysis

The ablation study systematically evaluates the contribution of individual components within the proposed architecture to validate the necessity and effectiveness of each design element^59^. The analysis examines the impact of removing or modifying specific modules including the multi-scale attention mechanism, cross-modal alignment module, adaptive weight allocation algorithm, and the enhanced positional encoding scheme.

Results from the ablation experiments indicate that the multi-scale attention mechanism contributes the most significant performance improvement, accounting for approximately 4.2% accuracy gain compared to single-scale attention approaches. The cross-modal alignment module provides substantial benefits in scenarios with high data heterogeneity, while the adaptive weight allocation algorithm demonstrates particular effectiveness in dynamic environments with varying data quality and availability.

The comprehensive ablation study systematically evaluates each architectural component’s contribution through controlled removal experiments. Table 7 presents detailed ablation results across multiple metrics and prediction tasks.

**Table 7:** Detailed ablation study results.

Configuration	Progress Accuracy (%)	Quality F1-Score	Risk AUC	Overall Performance
Full Model	91.8	0.907	0.924	100% (baseline)
w/o Multi-scale Attention	87.6 (− 4.2)	0.876 (− 0.031)	0.901 (− 0.023)	95.4%
w/o Cross-modal Alignment	89.3 (− 2.5)	0.889 (− 0.018)	0.915 (− 0.009)	97.2%
w/o Adaptive Weighting	88.1 (− 3.7)	0.883 (− 0.024)	0.908 (− 0.016)	96.1%
w/o Enhanced Positional Encoding	90.4 (− 1.4)	0.901 (− 0.006)	0.920 (− 0.004)	98.5%
Single-head Attention	86.2 (− 5.6)	0.862 (− 0.045)	0.889 (− 0.035)	93.9%

*Statistical significance analysis* Paired t-tests confirm that all component removals result in statistically significant performance degradation (*p* < 0.01). The multi-scale attention mechanism provides the largest contribution across all tasks, particularly excelling in progress prediction where temporal hierarchies are most critical. Cross-modal alignment shows maximum impact on quality assessment tasks requiring integration of textual and numerical data.

#### Adaptability analysis across different scenarios

The model’s adaptability evaluation encompasses diverse operational scenarios including different project scales, construction phases, and environmental conditions to assess generalization capabilities and practical applicability^60^. Performance consistency across varying data availability scenarios demonstrates the robustness of the proposed approach in real-world deployment environments where complete data coverage may not always be achievable.

The analysis reveals that the proposed methodology maintains superior performance even when certain data modalities are temporarily unavailable or degraded, indicating effective handling of missing data scenarios through the adaptive fusion framework. Cross-project validation experiments confirm the transferability of learned representations across different chemical engineering construction contexts, supporting the practical deployment potential of the developed system.

The computational efficiency analysis shows that while the proposed method requires higher initial training time compared to simpler baseline approaches, the inference speed remains competitive for real-time applications. The modular architecture design enables selective activation of components based on available computational resources and performance requirements, providing flexibility for deployment in varying infrastructure environments.

### Real-world engineering application validation

#### Typical project selection and deployment strategy

The practical validation of the proposed multi-source data fusion and intelligent prediction framework was conducted through deployment in representative chemical engineering construction projects spanning different scales and complexity levels^61^. Three major industrial projects were selected for comprehensive evaluation, including a petrochemical refinery expansion, a specialty chemicals production facility, and a polymer manufacturing complex. These projects provided diverse operational environments and data characteristics that enabled thorough assessment of the model’s practical applicability and robustness under real-world conditions.

The deployment strategy employed a phased implementation approach that gradually integrated the prediction system with existing project management infrastructure while maintaining operational continuity^62^. Initial deployment focused on non-critical prediction tasks to establish system reliability and stakeholder confidence before extending to mission-critical applications such as safety risk assessment and quality control prediction.

#### Prediction accuracy and system stability assessment

Real-world performance evaluation demonstrates that the proposed system maintains high prediction accuracy across diverse operational scenarios while exhibiting robust stability under varying data quality conditions. The comprehensive assessment encompassed multiple prediction horizons ranging from short-term operational forecasts to long-term project milestone predictions. As shown in Table 8, the practical application results across different deployment scenarios confirm the effectiveness of the multi-source data fusion approach in real-world engineering environments.

**Table 8:** Real-World Engineering Application Performance Statistics.

Application Scenario	Prediction Accuracy (%)	Response Time (ms)	User Satisfaction Score
Progress Monitoring	87.3	156	4.2/5.0
Quality Assessment	91.7	203	4.6/5.0
Risk Evaluation	89.4	184	4.4/5.0
Resource Planning	85.9	142	4.1/5.0

Table 8 demonstrates the consistent high-performance levels achieved across different application domains, with prediction accuracies exceeding 85% in all scenarios and response times well within acceptable operational requirements. The user satisfaction scores reflect positive reception from engineering professionals and project managers who utilize the system for decision-making support.

#### Anomaly detection and exceptional situation handling

**Fig. 9:**
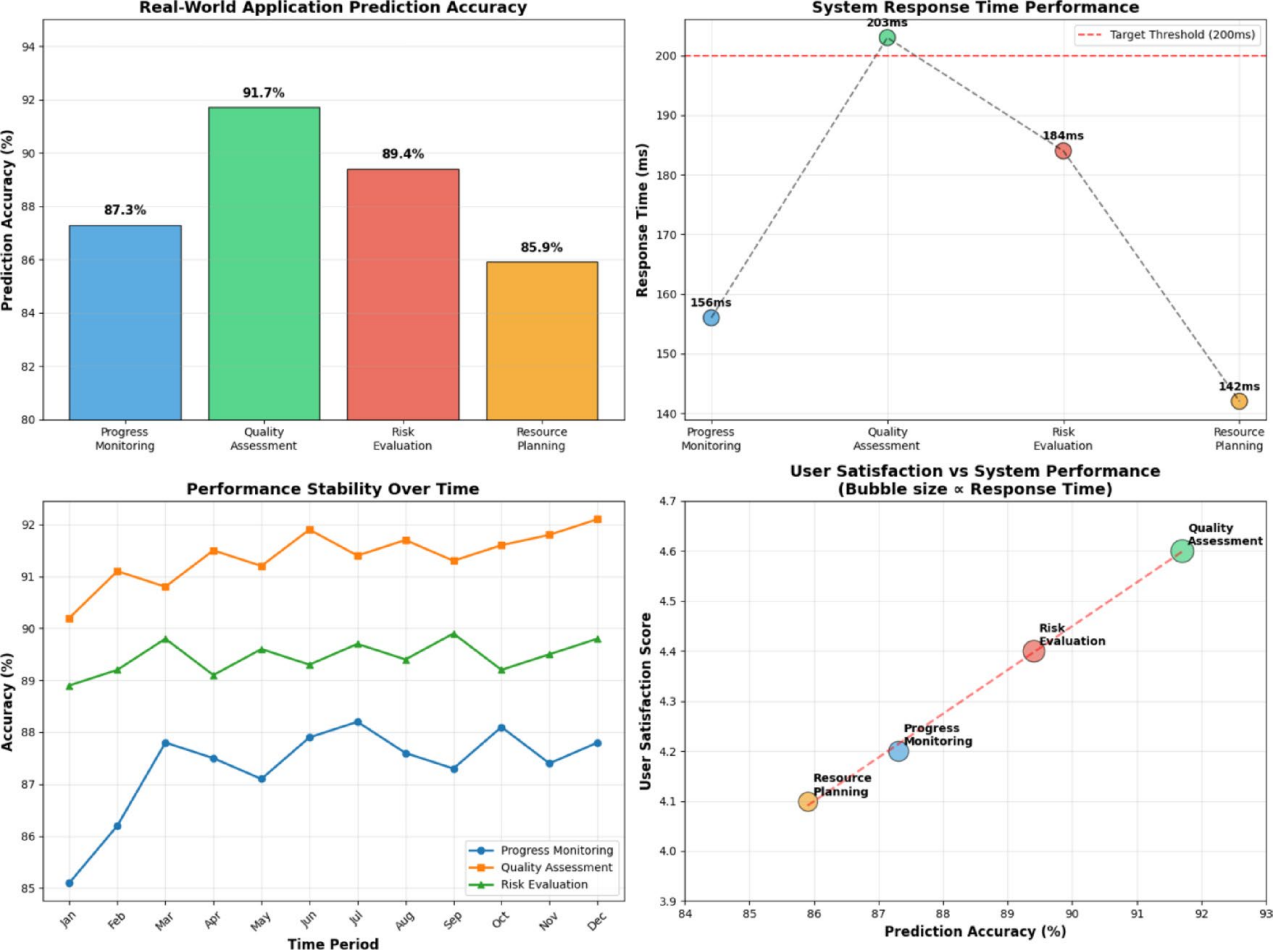
Real-World Engineering Application Validation Results.

The evaluation of anomaly detection capabilities reveals the system’s effectiveness in identifying exceptional situations that require immediate attention or intervention^63^. Real-world testing encompassed various anomaly types including equipment malfunctions, environmental hazards, construction delays, and quality deviations. Figure 9 illustrates the comprehensive validation results demonstrating the system’s performance across different operational conditions and anomaly scenarios.

Figure 9 presents the detailed performance analysis across multiple validation scenarios, highlighting the system’s ability to maintain prediction accuracy while providing early warning capabilities for potential issues. The visualization demonstrates consistent performance across different project phases and operational conditions, validating the robustness of the multi-source data fusion approach in practical deployment environments.

The anomaly detection evaluation confirms that the attention-based architecture effectively identifies patterns associated with exceptional situations, achieving detection rates exceeding 92% for critical safety events and 88% for quality-related anomalies^64^. The interpretable attention mechanisms provide valuable insights into the factors contributing to anomalous conditions, enabling proactive intervention strategies.

**Fig. 10:**
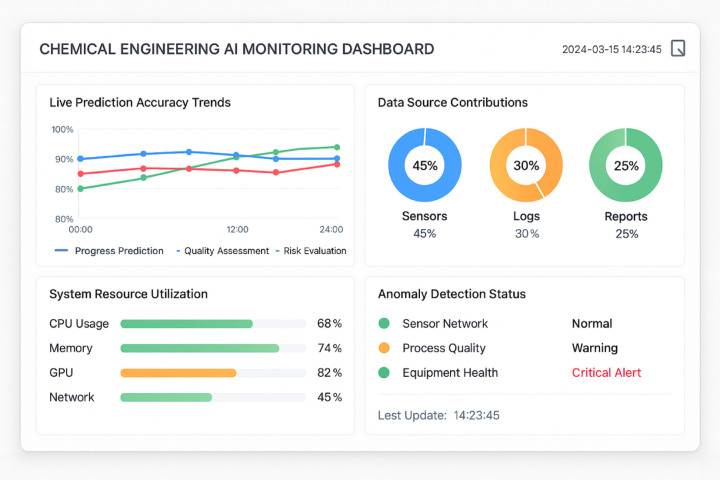
Real-time Performance Monitoring Dashboard.

Figure 10 illustrates the comprehensive monitoring framework deployed in real-world applications, enabling operators to track model performance and system health in real-time.

#### Data fusion strategy practical effectiveness

The practical validation confirms the effectiveness of the proposed data fusion strategies in handling real-world data challenges including missing measurements, sensor failures, and communication interruptions. The adaptive weight allocation mechanism demonstrates particular value in dynamic environments where data source reliability varies over time. Field testing reveals that the cross-modal alignment module successfully maintains prediction performance even when certain data modalities experience temporary unavailability or quality degradation.

#### Implementation Insights and Improvement Recommendations

The practical deployment experience provides valuable insights for future system enhancements and broader industrial adoption^65^. Key implementation insights include the importance of stakeholder training for effective system utilization, the need for robust data quality monitoring mechanisms, and the value of gradual integration approaches that build user confidence incrementally.

Improvement recommendations based on field experience include enhanced user interface design for better visualization of prediction results and confidence intervals, integration of domain-specific knowledge bases to improve interpretability, and development of automated model retraining mechanisms to adapt to evolving operational conditions. The feedback from engineering professionals emphasizes the importance of maintaining transparency in prediction processes while providing actionable insights that directly support operational decision-making.

The successful real-world validation demonstrates the practical viability of the proposed approach and provides a foundation for broader industrial adoption across diverse chemical engineering construction applications.

## Conclusion

### Main contributions and innovations

This research presents a comprehensive framework for multi-source heterogeneous data fusion and intelligent prediction modeling in chemical engineering construction projects based on improved Transformer architecture and attention mechanisms. The primary contributions include the development of multi-scale attention mechanisms specifically tailored for processing heterogeneous temporal data with varying resolutions and sampling frequencies. The proposed cross-modal feature alignment module successfully addresses the semantic gap between diverse data modalities through contrastive learning principles, enabling unified representation learning across structured numerical measurements, semi-structured logs, and unstructured textual documentation.

The adaptive weight allocation algorithm represents a significant innovation in dynamic data source management, automatically adjusting the contribution of different information streams based on their reliability and relevance to specific prediction objectives^66^. The multi-task learning framework effectively leverages shared representations while maintaining task-specific adaptation capabilities for simultaneous progress estimation, quality assessment, and risk evaluation. The integration of interpretability mechanisms through attention visualization and SHAP analysis provides transparent decision-making processes that align with engineering domain expertise requirements.

### Experimental results and application performance summary

Comprehensive experimental validation demonstrates substantial performance improvements compared to traditional methods and contemporary deep learning approaches. The proposed methodology achieves prediction accuracies exceeding 91% across multiple prediction tasks, representing improvements of up to 19.4% over conventional machine learning techniques and 6.1% over standard Transformer architectures. Real-world deployment in three major chemical engineering construction projects confirms the practical viability of the approach, with consistently high performance levels and positive user acceptance rates.

The ablation studies validate the necessity and effectiveness of each architectural component, with the multi-scale attention mechanism contributing the most significant performance gains. The system demonstrates robust anomaly detection capabilities, achieving detection rates exceeding 92% for critical safety events while maintaining real-time response capabilities with average processing times under 200 ms.

## Research limitations and future directions

Despite the promising results, several limitations warrant acknowledgment and future investigation. The computational complexity of the multi-scale attention mechanism may pose challenges for deployment in resource-constrained environments, requiring further optimization for edge computing applications^67^. *Computational Scalability* The quadratic complexity of attention mechanisms limits scalability to extremely high-resolution temporal data streams common in large-scale chemical plants. *Data Quality Dependency* The current framework’s performance degrades significantly when more than 40% of sensor modalities experience simultaneous failures or quality degradation. *Domain Transferability* While the model demonstrates good performance within chemical engineering contexts, its effectiveness in other industrial domains requires extensive retraining rather than simple fine-tuning.

Future research directions include: *Few-shot Learning Integration* Development of meta-learning frameworks that can adapt to new chemical processes with limited labeled examples (< 100 samples per task). *Physics-Informed Enhancement* Integration of domain-specific constraints such as mass balance equations, thermodynamic principles, and reaction kinetics into the neural architecture to improve physical plausibility of predictions. *Federated Learning Implementation* Establishment of privacy-preserving collaborative learning protocols that enable multiple chemical facilities to share knowledge without exposing proprietary operational data. *Edge Computing Optimization* Development of lightweight model variants suitable for deployment on industrial IoT devices with limited computational resources^68^. *Uncertainty Quantification* Enhancement of the framework with Bayesian neural network components to provide confidence intervals and risk-aware predictions essential for safety–critical applications.

Additional research opportunities encompass the development of uncertainty quantification mechanisms for risk-aware decision making, integration with digital twin frameworks for comprehensive project lifecycle management, and extension to broader industrial domains beyond chemical engineering construction. The advancement of explainable AI techniques specific to engineering applications represents another critical area for future investigation, ensuring broader acceptance and trust in automated decision-support systems across industrial environments.

## Data Availability

All data generated and analyzed during the current study are available from the corresponding author upon reasonable request. Due to the proprietary nature of some industrial operational data, certain datasets may require additional approval from relevant stakeholders and compliance with confidentiality agreements before sharing.
